# Double Negative B Cell Is Associated With Renal Impairment in Systemic Lupus Erythematosus and Acts as a Marker for Nephritis Remission

**DOI:** 10.3389/fmed.2020.00085

**Published:** 2020-04-07

**Authors:** Xujie You, Ruijun Zhang, Miao Shao, Jing He, Jiali Chen, Jiajia Liu, Xia Zhang, Xu Liu, Rulin Jia, Xiaolin Sun, Zhanguo Li

**Affiliations:** ^1^Department of Rheumatology and Immunology, Peking University People's Hospital, Beijing, China; ^2^Beijing Key Laboratory for Rheumatism and Immune Diagnosis (BZ0135), Peking University People's Hospital, Beijing, China

**Keywords:** double negative B cells, systemic lupus erythematosus, lupus nephritis, 24-h urine protein excretion, remission

## Abstract

**Objective:** Recent studies on double negative B cells (DN B cells) suggested that they have potential pathogenic roles in systemic lupus erythematosus (SLE). This study aimed to determine the circulating DN B cells in SLE patients and analyzed the clinical significance of this cell subset.

**Methods:** Fifty-seven SLE patients and fifty healthy controls (HCs) were recruited in this study. Among the 57 SLE patients, 25 had lupus nephritis (LN). All patients were followed up for 24 weeks. Peripheral B cell subsets were analyzed by flow cytometry.

**Results:** DN B cells were significantly elevated in the SLE patients, especially in the patients with LN (*p* < 0.01). DN B showed a positive correlation with 24-h urine protein excretion (24 h-UPE) levels (*r* = 0.444, *p* = 0.034) in LN patients, and inversely correlated with evaluated glomerular filtration rate (eGFR) (*r* = −0.351, *p* = 0.011). DN B cells had a positive correlation with plasma cells (*r* = 0.484, *p* < 0.001) and memory B cells (*r* = 0.703, *p* < 0.001). After treatment, decreased DN B cells were associated with LN alleviation (*p* = 0.002). In the follow-up, the remission rate of LN patients with decreased DN B cells was significantly higher than LN patients with increased DN B cells (83.33 vs. 25.00%, *p* = 0.030) at week 24.

**Conclusions:** This study suggests that the peripheral DN B cells are positively correlated with the severity of renal damage in LN patients and may potentially be used as a prognostic marker in LN.

## Introduction

Systemic lupus erythematosus (SLE) is a prototypic autoimmune disease with heterogeneous clinical manifestations that causes organ damage, and is triggered by a loss of self-tolerance, and autoreactive immune responses (1–3). Lupus nephritis (LN) is a frequent complication of SLE which can lead to significant illness and even death, despite great advances in treatment over the recent decades (4–7). The development of LN involves multiple pathogenic pathways, including inflammatory cell infiltration, autoantibody production, aberrant apoptosis, immune complex deposition, and complement activation (4, 8, 9). B cells are prominently involved in the pathogenesis of SLE and connect innate immunity with adaptive immunity, since they can both act as effector cells responding to antigens in humoral immunity and sense the environment and present autoantigens to T cells as antigen-presenting cells (10–12). Hyperactive polyclonal B cells could produce excessive pathogenic autoantibodies, cytokines, and chemokines (10, 13, 14).

The B cell compartment is severely unbalanced in patients with active SLE (15–18). In a previous study, Wei et al. described the expansion of B cells as characterized by lacking both IgD and CD27 (double negative; DN) in SLE and postulated that they represent a novel subset of memory cells (19). Double negative B cells (DN B cells) both in healthy subjects and SLE patients express switched and mutated antibodies. It has been proposed that DN B cells are involved in the pathogenesis of SLE. DN B cells were reported to be correlated with anti-dsDNA and anti-RNP/Sm autoantibodies (19–21). However, the detailed clinical relevance of DN B cells in SLE remains unclear.

In this study, we determined the circulating DN B cells in SLE patients and analyzed the clinical significance of this cell subset. Our study shows that DN B cells are positively correlated with 24-h urine protein excretion (24 h-UPE) in patients with lupus nephritis regardless of disease activity and decreased DN B cells are associated with renal alleviation. Thus, our finding suggests that DN B cells may be potential therapeutic targets for the treatment of lupus nephritis.

## Materials and Methods

### Patients and Healthy Controls

Patients with SLE satisfying the Systemic Lupus International Collaborating Clinics classification criteria were recruited from the Department of Rheumatology and Immunology in Peking University People's Hospital between February 2016 and January 2017. Fifty age-matched healthy controls (HCs) were enrolled. All participants were older than 16 years of age. All patients received standard-of-care therapy. The medications received by the patients were prednisone and other immunosuppressants, which were shown in Table S1. This study was approved by the ethics committee of Peking University People's Hospital. All patients provided informed consent to donate their blood samples and clinical information for research, and written consent was given by each individual.

### Flow Cytometry Analysis

Peripheral blood mononuclear cells (PBMCs) were isolated by Ficoll-Hypaque density gradient centrifugation. For surface staining, PBMCs were stained for 30 min in the dark at 4°C with the monoclonal antibodies Alexa Fluor 700 mouse anti-human CD3 (Biolegend), PE-CF594 mouse anti-human CD19 (BD Biosciences), PE mouse anti-human CD24 (eBioscience), APC mouse anti human CD27 (Biolegend), BV421 mouse anti-human CD38 (BD Biosciences), FITC mouse anti-human IgD (Biolegend), and PerCp-Cy5.5 mouse anti-human CD20 (Biolegend). Based different expression of surface markers, B cell subsets were identified: DN B cells (CD19+CD27–lgD–), memory B cells (CD19+CD27+), and plasma B cells (CD19+CD20–CD27+CD38+). Samples were examined on a BD FACS Aria II flow cytometer and data were analyzed by FlowJo version X.

### Clinical and Laboratory Evaluation

Clinical and laboratory data were collected at baseline, week 6, 12, and 24. Patient follow-ups were as scheduled by the treating physician. The following features of SLE were included in this study: rash, alopecia, photosensitivity, ulceration, myositis, fever, arthritis, leukopenia, thrombocytopenia, lupus nephritis, pericarditis, vasculitis, and NPSLE. Disease activity according to the Systemic Lupus Erythematosus Disease Activity Index-2000 (SLEDAI)-2K was recorded. A diagnosis of LN was made if patients fulfilled the American College of Rheumatology renal criteria (persistent proteinuria >0.5 g per day or >3+ by dipstick and/or cellular casts, including red cells, hemoglobin, granular, tubular, or mixed) at any time during the study. White cell and platelet counts <3.50 × 10^9^/L and 125 × 10^9^/L were regarded as leukocytopenia and thrombocytopenia, respectively. All patients underwent extensive serological examinations, including tests of antinuclear antibody (ANA), anti-dsDNA antibody (anti-dsDNA), anti-Sm antibody (anti-Sm), anti-Ro/SSA antibody (anti-SSA), anti-La/SSB antibody (anti-SSB), anti-RNP antibody (anti-RNP), complement component C3, complement component C4, erythrocyte sedimentation rate (ESR), C-reactive protein (CRP), immunoglobulin A (IgA), immunoglobulin G (IgG), and immunoglobulin M (IgM). C3, C4, IgA, IgG, and IgM were tested by ELISA with normal ranges of 0.79–1.52 g/L, 0.16–0.38 g/L, 0.82–4.53 g/L, 7.2–16.8 g/L, and 0.46–3.04 g/L. Anti-SSA and anti-SSB were measured by ELISA, and ANA was measured by indirect immunofluorescence. CRP was examined by immunonephelometry assays and values equal to or more than 8 mg/L were considered positive. Serum IFN-alpha2, IL-6, IL-2, IL-21, IL-7, IFN-gamma, IL-12p70, IL-15, IL-17A, BCA-1, IL-10, and TGF-beta were measured by ELISA. Complete renal remission (CR) in this study was defined as 24 h-UPE was <0.5 g/day and absence of hemoglobinuria or pyuria after therapy. Partly remissive (PR) LN was defined as a decrease of 24 h-UPE >50% after therapy.

### Statistical Analysis

SPSS 22.0 for windows and GraphPad Prism 7 were used to analyze the data. Data were presented as mean ± standard deviation and statistical significance between the two groups was assessed with the non-parametric Mann-Whitney test, paired *t*-test, and *X*^2^ test. Spearman's rank correlation coefficient was applied to calculate the correlations. Bonferroni correction was performed to adjust multiple comparisons in our correlation analysis. The Kaplan–Meier method was applied to compare the renal remission of the two groups during the follow-up. A value of *p* < 0.05 was considered to be significant.

## Results

### Characteristics of SLE Patients

In this study, 57 SLE patients and 50 healthy controls with matched demographic features (age, 30.96 ± 9.84 vs. 30.52 ± 5.66, *p* = 0.399; male/female, 4/53 versus 7/43, in SLE and HC, respectively, *p* = 0.386) were recruited. Detailed characteristics of SLE patients are shown in Table 1. The SLE patients had a mean disease duration of 62.43 months, ranging from 0 to 240 months and the mean SLEDAI of the patients was 11.74, ranging from 6 to 27 (Table 1).

**Table 1:** Demographic characteristics in patients with systemic lupus erythematosus.

**Clinical characteristics**	**Values**
Age, years	30.96 ± 9.84
Disease duration (months)	62.43 ± 63.13
SLEDAI-2K	11.74 ± 3.93
Gender (female: male)	53:4
Rash (%)	29/57 (50.88)
Arthritis (%)	27/57 (47.37)
Alopecia (%)	21/57 (36.84)
Lupus nephritis (%)	25/57 (43.86)
Pericarditis (%)	1/57 (1.75)
Thrombocytopenia (%)	12/57 (21.05)
Vasculitis (%)	6/57 (10.53)
Photosensitivity (%)	1/57 (1.75)
Ulceration (%)	5/57 (8.77)
Leukopenia (%)	3/57 (5.26)
Myositis (%)	3/57 (5.26)
Fever (%)	7/57 (12.28)
Headache (%)	2/57 (3.51)
Anemia (%)	19/55 (33.93)
Decreased C3 (%)	36/57 (63.16)
Decreased C4 (%)	35/57 (61.40)
Increased CRP (%)	12/53 (22.64)
Increased ESR (%)	24/56 (42.86)
ANA (+) (%)	42/45 (93.33)
Anti-dsDNA Ab (+) (%)	39/56 (69.64)
Anti-Sm (+) (%)	11/45 (24.44)
Anti-RNP (+) (%)	18/45 (40.00)
Anti-SSA (+) (%)	24/45 (53.33)
Anti-SSB (+) (%)	5/45 (11.11)
Proteinuria (+) (%)	37/55 (67.27)
Urine sediment erythrocytes (+) (%)	19/55 (34.55)
Urine sediment leukocytes (+) (%)	31/55 (56.36)

*SLEDAI-2K, systemic lupus erythematosus disease activity index 2000; LN, lupus nephritis; C3, complement 3; C4, complement 4; ESR, erythrocyte sedimentation rate; CRP, C-reactive protein; ANA, antinuclear antibodies; Anti-dsDNA Ab, anti-double-stranded DNA antibody; anti-Sm, anti-Sm antibody; Anti-RNP, anti ribonucleoprotein; Anti-SSA, anti-Ro/SSA antibody; anti-SSB, anti-La/SSB antibody. Numerical data presented as mean ± SD were analyzed using Student's t-test, Mann-Whitney U-test or Pearson's Chi-squared test. p <0.05 was taken as significant*.

### Circulating DN B Cells Increased in SLE Patients

**Figure 1:**
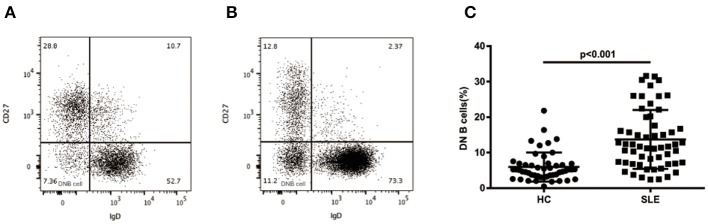
Surface phenotype of peripheral blood DN B cells. Dot plots represent staining of peripheral B cells in healthy subjects **(A)** and SLE **(B)** patients with increased frequency of DN B cells. **(C)** Comparative analysis of blood DN B cells from healthy controls and different groups of SLE patients. Higher levels of DN B cells were detected in SLE patients (*n* = 57) than in healthy subjects (*n* = 50). Differences compared to the HC were assessed by Mann-Whitney test.

As shown in Figures 1A,B, we evaluated the levels of CD19+CD27-IgD- DN B cells in PBMCs of HCs and SLE patients by flow cytometry. Compared with HCs, the SLE patients showed a significant increase of DN B cells (13.70 ± 8.28 vs. 5.95 ± 4.09%, *p* < 0.01; Figure 1C).

### Increased DN B Cells Were Correlated With Renal Involvement in SLE Patients

**Figure 2:**
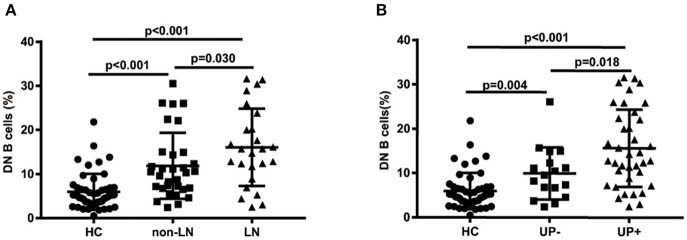
Comparative analysis of blood DN B cells from healthy controls and different groups of SLE patients. **(A)** Frequency of DN B cells in LN patients, non-LN patients, and healthy controls. Higher frequency of DN B cells was detected in LN patients (*n* = 25) than in non-LN patients (*n* = 32). **(B)** Comparison among healthy controls and SLE patients with proteinuria negative (*n* = 37) or positive (*n* = 18). Differences between groups are indicated. Differences were assessed by Mann-Whitney test.

**Figure 3:**
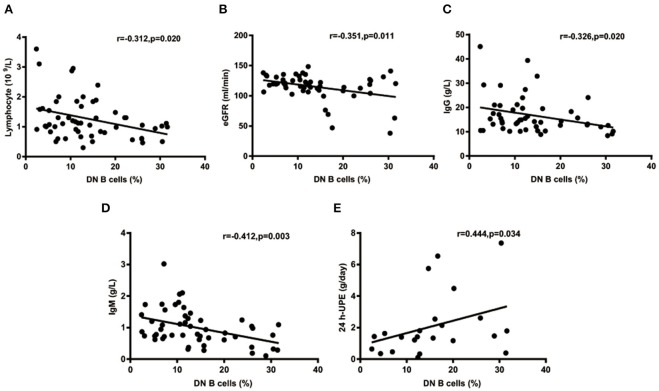
Correlations of circulating DN B cells in SLE with laboratory values. **(A–D)** DN B cells had an inverse correlation with Lymphocyte, eGFR, IgG, and IgM. **(E)** DN B cells had a positive correlation with 24 h-UPE. High DN B cells were associated with high 24 h-UPE in LN patients. *P* was calculated according to Spearman's correlation test.

The percentages of DN B cells were compared between patient groups with or without autoimmune clinical and laboratory features. Among the 57 SLE patients in our study (Table 1), 25 were diagnosed as LN. Compared with peripheral blood DN B cell levels of 11.86 ± 7.48 % in non-LN patients, increased peripheral blood DN B cell levels of 16.06 ± 8.79 % in LN patients were observed (*p* = 0.030; Table 2, Figure 2). We also found the subset from patients with urinary protein was increased significantly (*p* = 0.018; Table 2, Figure 2). These findings indicated an increase of DN B cell frequency in LN patients. We further analyzed the correlation between DN B cells and laboratory features of SLE. We found that DN B cells were positively correlated with 24 h-UPE levels (*r* = 0.444, *p* = 0.034) in LN patients (Table 3, Figure 3). The DN B cells had an inverse correlation with eGFR (*r* = −0.351, *p* = 0.011) in SLE patients. These results suggested that high levels of DN B cells were associated with impaired renal function.

**Table 2:** DN B cells in the presence or absence of clinical or laboratory characteristics.

**Characteristics**	**DN B cells**		***p*-value**
	**Presence (*n*)**	**Absence (*n*)**	
Rash	14.31 ± 9.26 (29)	13.07 ± 7.24 (28)	0.873
Arthritis	12.81 ± 7.33 (27)	14.51 ± 9.10 (30)	0.528
Alopecia	11.13 ± 7.04 (21)	15.20 ± 8.66 (36)	0.078
Lupus nephritis	16.06 ± 8.79 (25)	11.86 ± 7.48 (32)	**0.030**
Pericarditis	10.60 (1)	13.76 ± 8.34 (56)	0.761
Thrombocytopenia	14.86 ± 10.22 (12)	13.39 ± 7.79 (45)	0.845
Vasculitis	15.19 ± 6.82 (6)	13.53 ± 8.47 (51)	0.370
Photosensitivity	26.00 (1)	13.48 ± 8.18 (56)	0.181
Ulceration	13.64 ± 7.46 (5)	13.71 ± 8.42 (52)	0.855
Leukopenia	18.87 ± 10.24 (3)	13.41 ± 8.18 (54)	0.268
Myositis	8.94 ± 5.94 (3)	13.97 ± 8.35 (54)	0.292
Fever	12.68 ± 9.95 (7)	13.84 ± 8.12 (50)	0.451
Headache	10.82 ± 5.77 (2)	13.81 ± 8.37 (55)	0.696
Anemia	13.89 ± 8.77 (19)	13.74 ± 8.21 (37)	0.897
Decreased lymphocyte	15.96 ± 9.34 (28)	11.62 ± 6.64 (28)	0.103
Decreased C3	12.93 ± 7.28 (36)	15.02 ± 9.81 (21)	0.503
Decreased C4	12.67 ± 7.14 (35)	15.34 ± 9.77 (22)	0.342
Increased CRP	14.72 ± 9.53 (12)	13.15 ± 8.12 (41)	0.663
Increased ESR	15.03 ± 8.89 (24)	12.93 ± 7.84 (32)	0.362
ANA	12.98 ± 7.76 (42)	12.87 ± 2.47 (3)	0.716
Anti-dsDNA Ab	12.99 ± 8.04 (39)	15.82 ± 8.72 (17)	0.193
Anti-Sm	13.66 ± 6.39 (11)	13.66 ± 6.39 (34)	0.561
Anti-RNP	13.88 ± 9.10 (18)	12.36 ± 6.34 (27)	0.835
Anti-SSA	12.07 ± 6.39 (24)	13.99 ± 8.65 (21)	0.716
Anti-SSB	8.65 ± 4.65 (5)	13.51 ± 7.66 (40)	0.193
Proteinuria	15.41 ± 8.68 (37)	10.85 ± 6.83 (18)	**0.049**
Urine sediment erythrocytes	15.52 ± 8.63 (19)	13.08 ± 8.18 (36)	0.362
Urine sediment leukocytes	14.88 ± 8.91 (24)	13.18 ± 7.95 (31)	0.508

*C3, complement 3; C4, complement 4; ESR, erythrocyte sedimentation rate; CRP, C-reactive protein; ANA, antinuclear antibodies; Anti-dsDNA Ab, anti-double-stranded DNA antibody; anti-Sm, anti-Sm antibody; Anti-RNP, anti ribonucleoprotein; Anti-SSA, anti-Ro/SSA antibody; anti-SSB, anti-La/SSB antibody. P-value was determined by Mann-Whitney U-test. p <0.05 was taken as significant. The values in bold represent results with statistical significance*.

**Table 3:** Correlations of DN B cells with the laboratory parameters from SLE patients.

**Laboratory parameters**	**DN B cells**	
	***r***	***p*-value**
WBC	−0.114	0.402
Hb	−0.076	0.580
PLT	−0.020	0.886
Lymphocyte	−0.312	0.019*
eGFR	−0.351	0.011*
ALB	−0.132	0.358
ESR	0.054	0.692
CRP	0.132	0.347
IgA	0.053	0.712
IgG	−0.326	0.020*
IgM	−0.412	0.003*
C3	0.087	0.521
C4, g/L	0.232	0.082
Anti-dsDNA Ab	−0.061	0.654
SLEDAI-2K	0.088	0.516
24 h-UPE	0.444	0.034*
Urine sediment erythrocytes	0.227	0.096
Urine sediment leukocytes	−0.076	0.580

WBC, white blood cell; Hb, hemoglobin; PLT, platelet; eGFR, evaluated glomerular filtration rate; ALB, albumin; IgA, immunoglobulin A; IgG, immunoglobulin G; IgM, immunoglobulin M; C3, complement 3; C4, complement 4; CRP, C-reactive protein; ESR, erythrocyte sedimentation rate; Anti-dsDNA Ab, anti-double-stranded DNA antibody; SLEDAI-2K, systemic lupus erythematosus disease activity index 2000; 24 h-UPE, 24-h urine protein excretion. Correlation was examined by Spearman's rank correlation test. p <0.05 was taken as significant. The adjusted p-value is calculated by Bonferroni correction. The values with *represent not significant after correction for multiple comparisons. The values in bold represent results with statistical significance.

Since the difference in DN B cells between patients with LN and patients without LN might be associated with variances in the therapies, we summarized the treatments the patients received in Table S1. There was no significant difference in most of the treatments between SLE patients with LN and without LN. Compared with non-LN patients, a higher proportion of LN patients received azathioprine treatment (20 vs. 0%, *p* = 0.013; Table S1). To exclude the therapeutic influence, the percentages of DN B cells were further compared between LN patients treated with or without azathioprine, and no significant difference was observed (Figure S1, *p* = 0.362). These results suggested that the significant difference in DN B cell levels between LN and non-LN patients might not be induced by concurrent treatments.

### Correlation Between Plasma Cells or Memory B Cells and DN B Cell Subset in SLE Patients

**Figure 4:**
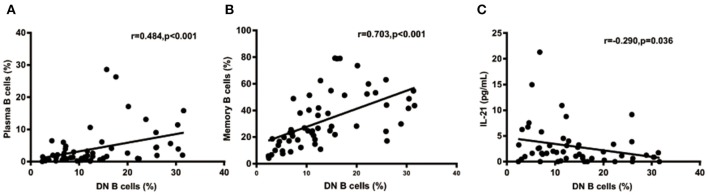
DN B cells in SLE patients are correlated with other subsets and cytokines. DN B cells had a positive correlation with plasma B cells **(A)** and memory B cells **(B)**. **(C)** An inverse correlation was observed between DN B cells and IL-21. *P* was calculated according to Spearman's correlation test.

We further analyzed the correlation between DN B cells and a variety of immune cell subsets in SLE. We found that DN B cells had a positive correlation with plasma cells (*r* = 0.484, *p* < 0.001) and memory B cells (*r* = 0.703, *p* < 0.001) (Figures 4A,B). No association was observed in naïve B cells, non-switched memory B cells, Treg, Tfh, Th1, Th2, Th17, and DN B cells. We also measured the concentration of serum cytokines in SLE patients. There was an inverse correlation between serum IL-21 and DN B cells (Table 4, Figure 4). DN B cells were also inversely correlated with lymphocyte (*r* = −0.312, *p* = 0.019), IgG (*r* = −0.326, *p* = 0.020), and IgM (*r* = −0.412, *p* = 0.003; Table 3, Figure 3). No association was observed between age or disease duration and DN B cells.

**Table 4:** Correlation analysis for serum cytokines and DN B cells.

**Cytokines**	**DN B cells**	
	***r***	***p*-value**
IFN-alpha2	−0.079	0.571
IL-6	−0.001	0.992
IL-2	−0.112	0.414
IL-21	−0.290	0.036*
IL-7	−0.257	0.058
IFN-gamma	−0.157	0.254
IL-12p70	0.046	0.740
IL-15	−0.258	0.060
IL-17A	−0.232	0.092
BCA-1	0.022	0.874
IL-10	−0.164	0.232
TGF-beta	0.141	0.313

Correlation was examined by Spearman's rank correlation test. p <0.05 was taken as significant. The adjusted p-value is calculated by Bonferroni correction. The value with *represents not significant after correction for multiple comparisons. The values in bold represent results with statistical significance.

### DN B Cell Decreases After Treatment Was Associated With LN Alleviation and Was a Prognostic Marker for Future LN Remission

In the follow-up, we further assessed the effect of DN B cells on LN. Among the 25 LN patients in our study, 19 LN patients had complete follow-up data. Patients with decreased 24 h-UPE were considered as responsive to therapy (responders, *n* = 12), and the others were regarded as failing to respond to therapy (non-responders, *n* = 7). According to the change trends of these subsets at week 6 after treatment, we divided LN patients into two groups: patients with increased DN B cells (Δ DN B cells > 0, *n* = 8) and patients with decreased DN B cells (Δ DN B cells <0, *n* = 11).

**Figure 5:**
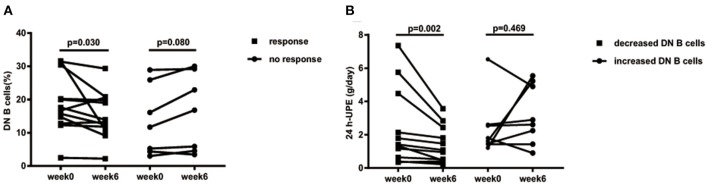
**(A)** Decreased 24 h-UPE in LN was accompanied by the decrease of DN B cells. Patients who had improvement at week 6 (*n* = 12) presented significantly decreased DN B cells. Those patients with a poor response (*n* = 7) showed a trend of increased DN B cells. **(B)** DN B cells correlated with 24 h-UPE in LN patients. 24 h-UPE decreased in LN patients with decreased DN B cells (*n* = 11). Differences were assessed by Wilcoxon test.

As the frequency of DN B cells was associated with LN involvement and 24 h-UPE level in LN patients, we speculate that effective therapy for LN might reduce the levels of DN B cells. Among LN patients under treatment, the percentages of DN B cells were indeed significantly decreased from 18.82 ± 8.727 to 15.26 ± 7.034% at week 6 in responsive LN patients (*n* = 12, *p* = 0.030; Figure 5A), whereas an obvious increase of DN B cells from 13.6 ± 10.51% at week 0 to 16.11 ± 11.61% at week 6 was observed in non-responsive patients (*n* = 7, *p* = 0.080).

The 24 h-UPE reduced from 2.44 ± 2.36 g/day at week 0 to 1.44 ± 1.11 g/day at week 6 in LN patients with decreased DN B cells (*p* = 0.002; Figure 4B); however an increase of 24 h-UPE was found (2.4 ± 1.75 g/day at week 0 vs. 3.22 ± 1.78 g/day at week 6) in LN patients with increased DN B cells (*p* = 0.469; Figure 5B).

**Figure 6:**
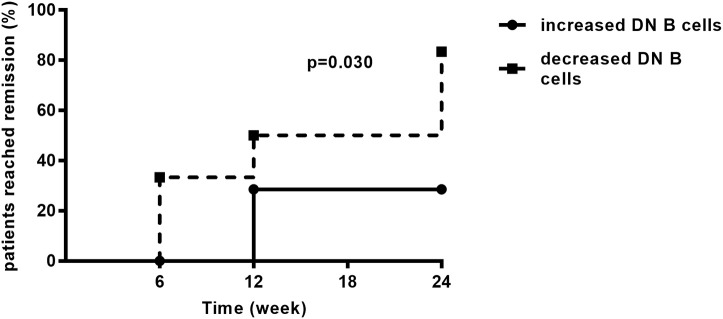
During the follow-up, LN patients achieved remission. The remission rate was higher in LN patients with decreased DN B cells (*n* = 11) than those with increased DN B cells (*n* = 8). Differences were assessed by Kaplan–Meier method.

As mentioned above, LN patients were divided into two groups according to the changes of DN B cells at week 6. During the follow-up period of 24 weeks, the remission rate in the group of patients with decreased DN B cells was 81.82%, which was significantly higher than in the other group (25.00%, *p* = 0.030; Figure 6).

## Discussion

Conventional memory B cells can be distinguished from naïve B cells by the presence of somatic hypermutation in their Ig variable gene sequences (22). CD27 is generally used as a marker to identify memory B cells because its expression correlates with the presence of somatic mutations in Ig genes (23, 24). However, further research elucidated that between 10 and 20% of IgG+ class-switched memory B cells were CD27- (25). Absence of IgD expression on DN B cells indicates that they have undergone class switching, though they do not gain the expression of CD27. DN B cells could represent a novel population of memory cells lacking CD27 expression (19). Early studies demonstrated that DN B cells can be detected in healthy persons, but are expanded in elderly people (26), patients with systemic lupus erythematosus, rheumatoid arthritis (27), Alzheimer's disease (28), non-small cell lung cancer (29), rotavirus (30), and HIV (31). Previous work showed that DN B cells are expanded in SLE patients (19). However, its clinical relevance in SLE is not yet clearly understood.

Our study demonstrated that LN patients had significantly higher DN B cells than non-LN patients, suggesting that DN B cells are involved in the renal damage associated with SLE. This result is consistent with previous studies that showed DN B cells expanded in patients with LN (16, 21). In addition, the levels of DN B cells were positively correlated to 24 h-UPE, suggesting that DN B cells may be involved in the severity of renal damage in LN patients. Consistent with this notion, the kidney damage of LN patients was effectively alleviated by drug treatment, accompanied by the downregulation of DN B cells. This result raised the possibility that DN B cells might be used as a prognostic marker in LN. Previous studies indicate that DN B cells are a heterogeneous subpopulation in SLE patients and DN B cells in the peripheral did not correlate significantly with disease activity (32). Our results were in agreement with this study and no correlation between DN B cells and SLEDAI was observed. The relationship between DN B cells and SLE is likely to be complex. Our data presents new evidence that DN B cells may play pathogenic roles in LN specifically. In previous studies, transcriptomic analysis on DN B cell subsets showed altered expression of multiple chemokine receptors including CCR9, CCR7, and CXCR5. In SLE patients, the alteration of the chemokine signaling network might lead to the chemotaxis of DN B cells into inflammatory tissues, like kidneys, to aggravate the local inflammation. These studies also showed that DN B cells were prone to differentiate into plasma cells, which preferentially produced pathogenic autoreactive antibodies in SLE. Altogether, these reports indicate that DN B cells might migrate to the relevant renal tissues and play a pathogenic role either *in situ* in the kidney or by their active production of autoantibodies.

We found that DN B cells correlated with decreased lymphocytes or eGFRs, which are associated with SLE. As we know, lymphopenia is a typical feature of SLE (33–35). Glomerular filtration rate (GFR) is one of the conventional clinical parameters for detecting ongoing disease activity in lupus-affected kidneys and early relapse of nephritis (36, 37). A low GFR is a dangerous sign of existing kidney disease. Our data presented more evidence that DN B cells were specifically associated with LN development. Previous studies (20, 38) showed that DN B cells were correlated with anti-dsDNA or anti-RNP/Sm autoantibodies, but our study didn't observe such an association. It is possible that the limited cohort size in our study led to the lack of statistical significance. Differences in autoantibody testing technology or genetic background of recruited patients between previous studies and this study might also be possible reasons for this discrepancy.

IL-21 plays a critical role in B-cell differentiation and antibody production (39). Previous studies showed that IL-21 increased the number of transitional B cells, post-switched memory B cells, and plasma cells and promoted serum IgG and IgM production (40, 41). In our study, an inverse correlation was found between serum IL-21 and DN B cells. This may be explained by the possibility that DN B cells consumed IL-21 to differentiate into plasma cells. Our results also showed that increased levels of DN B cells were associated with elevated levels of plasma cells, which also supported the above hypothesis. These results suggest that DN B cells might play a role in the enhanced humoral immune response in SLE. However, the main limitation on our hypothesis is the lack of experimental evidence in this study. Previous studies have shown that the IL-21 receptor (IL-21R) was expressed at high levels in DN B cells, and after binding IL-21 with IL-21R, DN B subsets efficiently differentiated into plasma cells. These studies could provide some supportive experimental clues to our hypothesis.

During the 24-weeks follow-up, the remission rates of LN patients with decreased DN B cells and increased DN B cells at week 6 were 83.33 and 25.00% (*p* = 0.030), respectively. These results demonstrate that patients with decreased DN B cells under treatment are likely to achieve renal remission, and this is the first study showing that DN B cells might be a prognostic marker in LN.

One limitation of this study is that the SLE patients received different treatments during the study. Since therapy could affect the immune phenotypes, it is possible that the difference in DN B cells between patients with LN and patients without LN is associated with the possible variances in the treatments they received. Our analysis showed that there was no significant difference in most of the treatments between LN patients and non-LN patients. Although 20% of LN patients received azathioprine treatment while non-LN patients did not, no difference in the percentages of DN B cells were observed between LN patients treated with or without azathioprine. These results suggest that different treatments might not lead to different DN B cell levels in LN patients and non-LN patients in the current study. However, it is still possible that differences in DN B cells might be induced by different therapies, and future studies with a larger cohort size should be performed to elucidate the possible effects of different treatments on DN B cells.

Another limitation of this study is shown by the adjusted *p*-value of correlation analysis with Bonferroni correction (Tables 3, 4). After the correction, no statistical significance was observed in the adjusted *p*-values. It is possible that the limited cohort size in our study led to the lack of statistical power when correction for multiple comparison was performed.

This study suggests that DN B cells correlate with the severity of renal damage in LN patients. Also, DN B cells may be involved in the pathogenesis of LN. Furthermore, decreased DN B cells are associated with renal alleviation during the follow-up. Specifically, our findings indicate that DN B cells may be used as a prognostic marker in LN.

## Data Availability Statement

The datasets generated for this study are available on request to the corresponding author.

## Ethics Statement

This study was approved by the ethics committee of Peking University People's Hospital. All patients provided informed consent to donate their blood samples and clinical information for research, and written consent was given by each individual.

## Author Contributions

XY performed the experiment and statistical analysis and wrote the draft of the manuscript. ZL and XS designed the study, analyzed the data, and wrote the manuscript. RZ and MS participated in acquiring clinical data and performed some experiments. JH, JC, JL, XZ, XL, and RJ participated in the analyses. All authors contributed to manuscript revision and have read and approved the submitted version.

### Conflict of Interest

The authors declare that the research was conducted in the absence of any commercial or financial relationships that could be construed as a potential conflict of interest.
