# Supplementary material for: Double Negative B Cell Is Associated With Renal Impairment in Systemic Lupus Erythematosus and Acts as a Marker for Nephritis Remission
Source: Front Med (Lausanne). 2020 Apr 7;7:85. doi: 10.3389/fmed.2020.00085 (PMC7155774; doi:10.3389/fmed.2020.00085)
Supplement: Supplementary file 1 [file Data_Sheet_1.docx]

Table S1 Medications of SLE patients in this study.

| Medications | LN patients | Non-LN patients | p value |
| --- | --- | --- | --- |
| Prednisone dose, mg/day, median (range) | 12.5(7.5,50) | 15(0,50) | 0.800 |
| Hydroxychloroquine | 24(96.00) | 32(1) | 0.439 |
| Cyclophosphamide | 2(8) | 1(3.13) | 0.576 |
| Azathioprine | 5(20.00) | 0(0) | **0.013** |
| Cyclosporine A | 1(4.00) | 4(12.50) | 0.372 |
| Mycophenolate Mofetil | 9(36) | 9(28.13) | 0.575 |
| Tacrolimus | 2(8) | 0(0) | 0.188 |
| Leflunomide | 2(8) | 2(6.25) | 1 |
| Thalidomide | 0(0) | 1(3.13) | 1 |
| Methotrexate | 0(0) | 2(6.25) | 0.499 |

SLE: systemic lupus erythematosus; LN: Lupus nephritis. P value was determined by Mann-Whitney test and Pearson’s Chi-squared test. p< 0.05 was taken as significant.

Figure S1


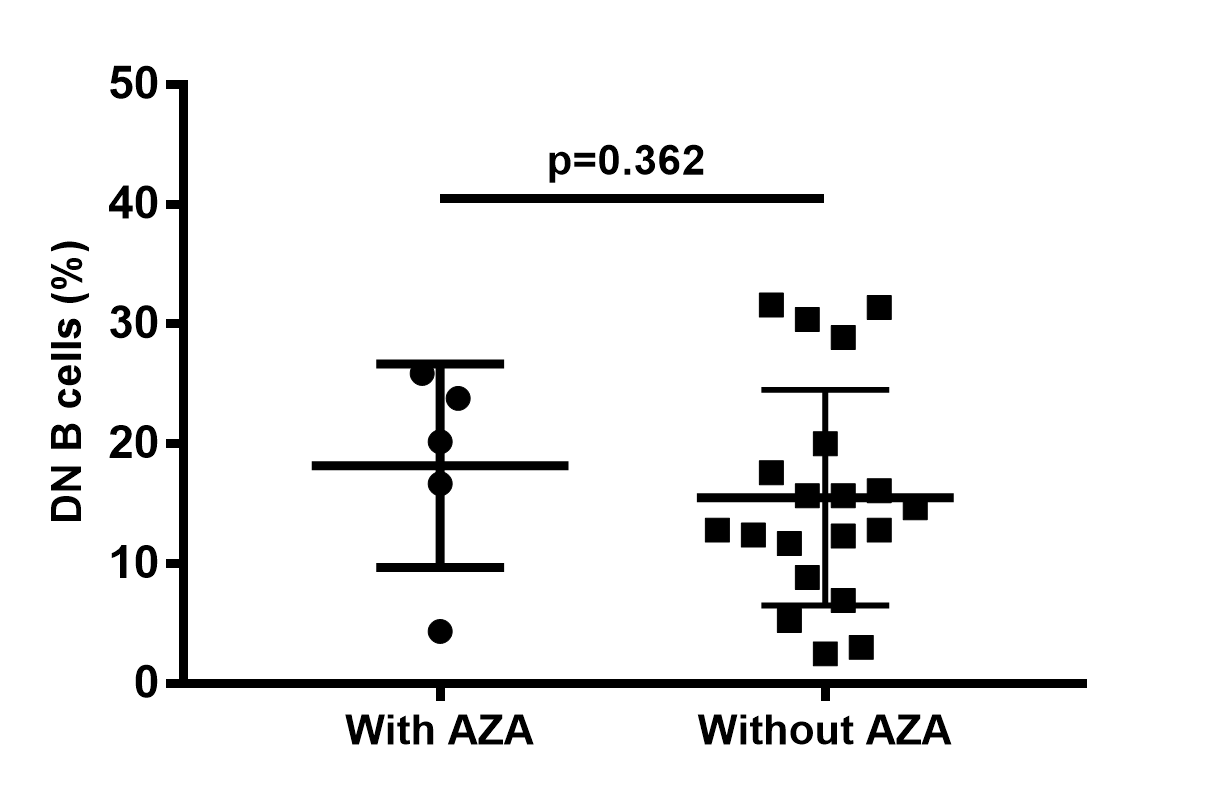


Figure S1 Comparative analysis of blood DN B cells from LN patients treated with or without azathioprine. P was calculated according to Mann-Whitney test. LN: Lupus nephritis; AZA: azathioprine.
